# THE POTENTIAL OF CROWDSOURCED ASSET MAPPING TECHNOLOGIES FOR SUPPORTING DEMENTIA CAREGIVERS

**DOI:** 10.1093/geroni/igad104.1753

**Published:** 2023-12-21

**Authors:** Nicole Ruggiano, Tish Winton, Jane Daquin, Zhe Jiang, Monica Herzog, Jeff Gray

**Affiliations:** University of Alabama, Hoover, Alabama, United States; University of Alabama, Tuscaloosa, Alabama, United States; University of Alabama, Tuscaloosa, Alabama, United States; Computer & Information Science & Engineering, Gainsville, Florida, United States; College of Engineering, Tuscaloosa, Alabama, United States; University of Alabama, Tuscaloosa, Alabama, United States

## Abstract

It is well-established that caregivers of people living with dementia (PLWD) often report having difficulty finding information about education and support that is available to them in their community. Caregiver support groups have demonstrated to be an effective approach to caregiver education through mutual support, though many caregivers are unable to participate in caregiver support groups due to time and geographic constraints. Crowdsourced technologies that rely on volunteered geographic information (VGI) are a viable way of community members to provide mutual support on a variety of issues, including disaster response, traffic conditions, health, and travel. These technologies often rely on a mobile app where users upload VGI that are of interest to others who use the app. There are benefits and drawbacks of crowdsourced technologies. For example, they may be an easy way for community members to share needed information, though there may be challenges in the quality of data that users provide. This presentation will report findings from a project that examined dementia caregivers’ interest in using crowdsourced mapping technologies to share information about services and resources in their community that they have found helpful. Data were collected from a diverse sample of dementia caregivers living in Alabama through an online survey that was administered between June and November of 2022. Overall, it was found that caregivers were interested in using a crowdsourcing technology for support and education. Additional findings provide guidance for developing a crowdsourcing protocol that is based on caregivers’ reported information needs.

